# Stakeholder engagement in healthcare research in India – A systematic review

**DOI:** 10.1186/s12961-025-01341-9

**Published:** 2025-05-15

**Authors:** Remya U. Rajendran, Baby S. Nayak, N. Siva, Tenzin Phagdol, Mamatha Shivananda Pai, Preethy D’Souza, Judith Angelitta Noronha

**Affiliations:** 1 Department of Child Health Nursing, Manipal College of Nursing, Manipal Academy of Higher Education, Manipal, Karnataka India; 2 Department of Child Health Nursing, SUM Nursing College, Siksha ‘O’ Anusandhan (SOA) University, Bhubaneswar, Odisha India; 3 Social Research Institute, Institute of Education, University College London, London, United Kingdom; 4 Department of Obstetrics&Gynaecological Nursing, Manipal College of Nursing, Manipal Academy of Higher Education, Manipal, Karnataka India

**Keywords:** Stakeholder, Engagement, Approaches, Healthcare, Research, India, Health equity, Child health, Reproductive health, Mental health, Public health, Universal health coverage, Preventable diseases, Primary health nursing

## Abstract

**Background:**

Stakeholder engagement is increasingly crucial in healthcare research, particularly in diverse and complex settings such as India. Stakeholder engagement in health research is about collaborating with key parties such as patients, healthcare providers and policymakers to ensure the research is relevant and impactful by addressing real-world needs, thereby enhancing its quality and effect on healthcare practices.

**Aim:**

The purpose of this study was to summarize the evidence on stakeholder engagement in healthcare research and its influence on research outcomes and healthcare policies in India.

**Methods:**

The evaluation was conducted following the Preferred Reporting Items for Systematic Reviews and Meta-Analyses guidelines. A systematic search was conducted in PubMed, SCOPUS, ProQuest, EMBASE, Web of Science, CINAHL Indian Citation Index and J-Gate, focussing on stakeholder involvement in healthcare settings in hospitals and communities in India. Various research methodologies were employed, with studies not centred on healthcare stakeholder engagement or unrelated sectors being excluded. Tools such as the Critical Appraisal Skills Programme checklist for qualitative studies and the mixed methods appraisal tool were used to evaluate the quality of the studies. Data synthesis was carried out using the descriptive/narrative synthesis approach.

**Results:**

We included 25 articles on the basis of our eligibility criteria. These articles comprised reviews, theories of change, quantitative studies, reports, mapping, commentaries, conference proceedings, qualitative studies, experience papers and mixed methods research. The review examined different types and methods of engaging stakeholders in healthcare research projects, evaluated their influence on evidence-based practice, and investigated their relevance to reaching “hard-to-reach” populations. Overcoming financial, time, knowledge and logistical barriers and gaining support from international and governmental bodies can lead to more inclusive research with a significant impact.

**Conclusions:**

Findings suggest that stakeholder engagement contributes to more contextually relevant and ethically grounded research, though challenges related to power dynamics, resource allocation and inclusivity remain prevalent. The review concludes by providing recommendations for enhancing stakeholder engagement practices in future healthcare research in India, emphasizing the need for capacity-building and inclusive frameworks that ensure diverse voices are represented.

## Introduction

Stakeholders in healthcare, defined as individuals or groups responsible for or impacted by health-related decisions influenced by research evidence, encompass a diverse range of entities, including patients, caregivers, families, advocacy organizations, healthcare providers, payers, purchasers, policymakers, product manufacturers, researchers and the press [1–4]. The growing emphasis on protocols that involve diverse stakeholders, particularly in patient-centred care policies, highlights the increasing importance of stakeholder engagement in healthcare research [1, 3]. Researchers must identify and involve key stakeholders across various healthcare system levels, including policymakers, healthcare providers and community healthcare workers [4–6].

India’s healthcare system faces several significant challenges, including a high out-of-pocket expenditure, with nearly 75% of healthcare costs borne by individuals, leading to the financial burden on households. Furthermore, issues such as limited access to quality care, especially in rural areas, and a shortage of trained medical professionals, worsen the situation [7]. The quality of care in India’s healthcare sector is inconsistent, ranging from internationally recognized institutions to facilities that offer inadequate services. With the rise of chronic conditions, government and nongovernment research institutes, researchers and policymakers must collaborate to enhance healthcare quality and implement evidence-based initiatives [8–10]. Identifying key stakeholders and implementing successful healthcare initiatives are essential for offering continuous patient support, tailored education and enhancing healthcare providers’ capacity to address challenges in complex situations [11, 12]. As efforts to improve care quality grow, public and private sectors address issues with data reliability and measurement complexities, prioritizing accuracy enhancements, refining methodologies and seeking innovative solutions [8, 13]. The effectiveness of stakeholder engagement in research is influenced by structural, cultural and individual practices, affecting its practical implementation [4, 14].

Healthcare research has evolved from a solitary scientist approach to a more inclusive model, emphasizing multidisciplinary team science [15]. Recent advancements involve actively engaging stakeholders in various medical research activities [16]. Their involvement focusses on building shared understandings, which are crucial for accepting and implementing recommendations rather than just practical reasons [17]. There is a significant gap in the existing literature regarding the lack of systematic approaches for implementing sustainable stakeholder engagement in healthcare research, especially in ensuring long-term impacts on care quality and integration within healthcare systems. On the basis of existing evidence, researchers conducted a systematic review to uncover stakeholders’ diverse roles and responsibilities, demonstrating their contributions to healthcare research. The findings highlight the feasibility and potential adoption of stakeholder engagement at different levels of healthcare research whilst also revealing various approaches to implementing sustainable stakeholder engagement to improve care quality within healthcare systems.

### Review question

1. What are the different approaches used for stakeholder engagement in healthcare research?
2. How do stakeholders influence research outcomes and healthcare policy development in India?

*Aim* To collate and summarize the evidence on various stakeholder engagement approaches used in healthcare research and its role in influencing research outcomes and shaping healthcare policies in India.

## Methods

### Search strategy

The findings of this systematic review were reported by the Preferred Reporting Items for Systematic Review and Meta-Analysis (PRISMA) guidelines [18]. The PRISMA checklist is available in Additional file 1.

A comprehensive search was conducted to find the primary articles in six international databases: PubMed, SCOPUS, ProQuest, EMBASE, Web of Science and CINHAL, using specific keywords. Additionally, we conducted in-depth searches in Indian databases such as the Indian Citation Index and J-Gate. In addition, institutional and non-institutional repositories, including Shodhganga, the National Institute of Science Communication and Information Resources and the Indian Institute of Science’s ePrints@IISc, were examined. Relevant publications were also retrieved from the Campbell Collaboration, the Public Health Foundation of India (PHFI), the WHO, the Indian Institute of Public Health and Indian research funding agencies. Further resources included the Indian Science Abstracts (ISA) and Semantic Scholar. Finally, reference lists of included studies and related systematic reviews were screened for any additional pertinent studies.

The search criteria included specific keywords such as stakeholder OR stakeholder* OR expert* OR collaborator* OR “health professional” OR “health care provider*” OR “community health personnel” OR"Health Services Research"OR “health care” OR “health facility*” OR “health service*” OR “health research”. participant* OR participation OR “stakeholder engagement” OR “stakeholder involvement” OR “stakeholder role*” AND research OR “health research” OR healthcare OR “quality of care” OR “healthcare system” OR Improvement (Table 1).

**Table 1:** Search strategy used for six databases

Database	Search string
PubMed	(“stakeholder”[Title/Abstract] OR “stakeholders”[Title/Abstract] OR “expert*”[Title/Abstract] OR “collaborator*”[Title/Abstract] OR “health professional”[Title/Abstract] OR “health care provider*”[Title/Abstract] OR “community health personnel”[Title/Abstract]) AND (“Health Services Research”[MeSH] OR “health care”[Title/Abstract] OR “health facility*”[Title/Abstract] OR “health service*”[Title/Abstract] OR “health research”[Title/Abstract]) AND (“participant*”[Title/Abstract] OR “participation”[Title/Abstract] OR “stakeholder engagement”[Title/Abstract] OR “stakeholder involvement”[Title/Abstract] OR “stakeholder role*”[Title/Abstract]) AND (“research”[Title/Abstract] OR “health research”[Title/Abstract] OR “healthcare”[Title/Abstract] OR “quality of care”[Title/Abstract] OR “healthcare system”[Title/Abstract] OR “improvement”[Title/Abstract])
SCOPUS	TITLE-ABS-KEY(“stakeholder” OR “stakeholder*” OR “expert*” OR “collaborator*” OR “health professional” OR “health care provider*” OR “community health personnel”) AND TITLE-ABS-KEY(“Health Services Research” OR “health care” OR “health facility*” OR “health service*” OR “health research”) AND TITLE-ABS-KEY(“participant*” OR “participation” OR “stakeholder engagement” OR “stakeholder involvement” OR “stakeholder role*”) AND TITLE-ABS-KEY(“research” OR “health research” OR “healthcare” OR “quality of care” OR “healthcare system” OR “improvement”)
ProQuest	(“stakeholder” OR “stakeholder*” OR “expert*” OR “collaborator*” OR “health professional"OR “health care provider*” OR “community health personnel”) AND (“Health Services Research” OR “health care” OR “health facility*” OR “health service*” OR “health research”) AND (“participant*” OR"participation” OR “stakeholder engagement” OR “stakeholder involvement"OR “stakeholder role*”) AND (“research” OR “health research"OR “healthcare” OR “quality of care"OR “healthcare system” OR “improvement”)
EMBASE	(“stakeholder”/exp OR stakeholder* OR expert* OR collaborator* OR “health professional”/exp OR “health care provider*” OR “community health personnel”/exp) AND (“health services research”/exp OR “health care”/exp OR “health facility*” OR'health service*” OR “health research”/exp) AND (“participant*” OR “participation” OR “stakeholder engagement” OR “stakeholder involvement” OR “stakeholder role*”) AND ('research”/exp OR “health research”/exp OR “healthcare”/exp OR “quality of care”/exp OR “healthcare system”/exp OR “improvement”/exp)
Web of Science	TS = ((stakeholder OR stakeholder* OR expert* OR collaborator* OR “health professional” OR “health care provider*” OR “community health personnel”) AND (“Health Services Research” OR “health care” OR “health facility*” OR “health service*” OR “health research")) AND (participant* OR participation OR “stakeholder engagement” OR “stakeholder involvement” OR “stakeholder role*”) AND (research OR “health research” OR healthcare OR “quality of care” OR “healthcare system” OR improvement)
CINAHL	(MH “Stakeholder Engagement” OR stakeholder OR stakeholder* OR expert* OR collaborator* OR “health professional” OR “health care provider*” OR “community health personnel”) AND (MH “Health Services Research” OR “health care” OR “health facility*” OR “health service*"OR “health research") AND (participant* OR participation OR “stakeholder engagement” OR “stakeholder involvement"OR “stakeholder role*”) AND (research OR “health research” OR healthcare OR “quality of care” OR “healthcare system” OR improvement)

### Inclusion criteria

The review included comprehensive primary research carried out in both hospital and community settings within the healthcare domain, providing empirical evidence on stakeholder involvement in healthcare research across India. The type of studies included in the review were various research methodologies such as observational, cross-sectional, descriptive, qualitative and mixed-method studies, alongside review articles, commentaries and conference proceedings emphasizing evidence generation and synthesis in healthcare research. Excluded were studies not specifically addressing stakeholder engagement in healthcare research within the Indian context, as well as those exploring stakeholder involvement in sectors unrelated to healthcare research.

### Screening process

Database searches were exported to Rayyan software, and title and abstract screening were conducted independently by two reviewers (R.U.R. and N.S.). Following this, full-text screening was carried out for studies deemed eligible for inclusion in the review. An independent appraisal of studies was conducted by two reviewers, who assessed the studies separately to minimize bias and ensure objectivity. In cases where disagreements arose, a third reviewer was consulted, and consensus was reached on the basis of predefined criteria, including study relevance to the research question, adherence to inclusion/exclusion criteria and methodological quality. This approach ensured a transparent and systematic selection process, maintaining the rigour and reliability of the review. Finally, selected studies were included in the review and subjected to quality assessment and data extraction.

### Quality assessment

The Critical Appraisal Skills Programme (CASP) checklist for qualitative studies [19] and the mixed methods appraisal tool (MMAT) checklist for mixed methods studies [20] were used to assess the quality of the included studies. The CASP qualitative checklist consists of two screening questions (yes/no) and eight additional questions (yes/no/cannot tell) if both questions receive a “yes” response. As described by Long and French, study quality was assessed based on the rigour of data analysis and the trustworthiness of the results. Considering these factors alongside the overall checklist score, studies were categorized as high, moderate or lower quality [21]. The mixed methods appraisal tool (MMAT) helps to identify whether the study is qualitative (mapping &theory of change), quantitative (descriptive, non-randomized, randomized) or mixed methods using MMAT’s classification and answering five key questions rated yes, no or can’t tell. A study meeting all five criteria is considered high quality, whilst one failing multiple criteria may be moderate or low [20].

### Data extraction

Two researchers (R.U.R. and N.S.) conducted data extraction using Excel sheets from various sources. The data we collected includes specific details about the state and country where the studies took place, the titles and objectives of the studies, the samples and sample sizes used and the study designs or methods used. We also noted the approaches or strategies for engaging stakeholders, the types of stakeholders involved, and a detailed description of their roles in each study. We documented the data collection methods, including the tools and techniques used and the statistical analysis approaches employed to interpret the findings. We extracted the overall findings of the studies and recorded information about the funding sources that supported the research.

## Results

### Study selection

**Fig. 1:**
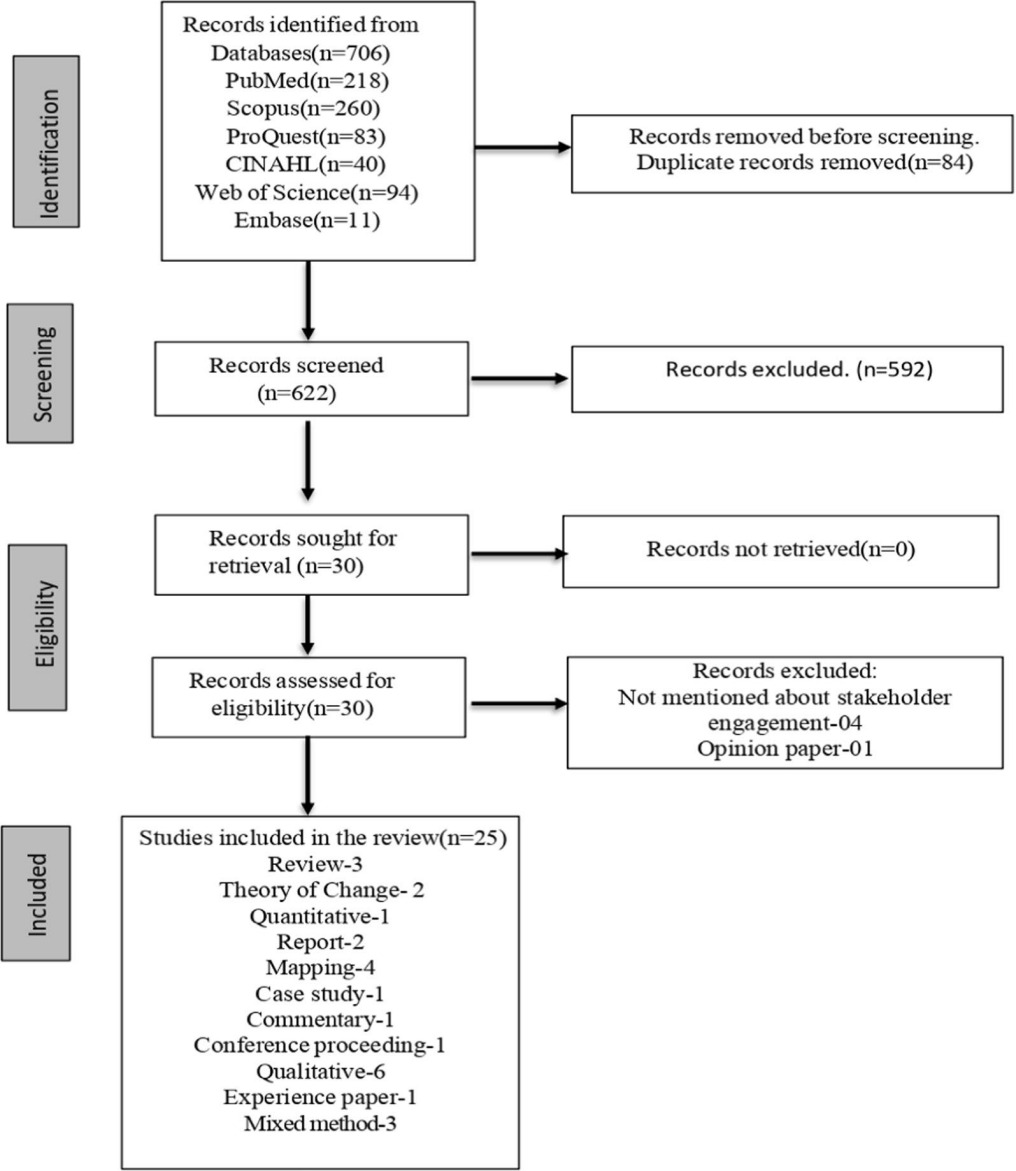
PRISMA flow diagram

We initially identified 706 records. After 84 duplicates were removed, 622 articles underwent title and abstract screening. Following the inclusion criteria, 592 studies were excluded. Subsequently, we reviewed the full texts of 30 studies. Finally, 25 articles were included, comprising review (*N* = 3), theory of change (*N* = 2), quantitative (*N* = 1), report (*N* = 3), mapping (*N* = 4), commentary (*N* = 1), conference proceeding (*N* = 1), qualitative (*N* = 6), experience paper (*N* = 1) and mixed method (*N* = 3). The study selection process is illustrated in the PRISMA flow diagram in Fig. 1, whilst Table 2 outlines the characteristics of the included studies.

**Table 2:** Characteristics of included studies

Author and year	Area of research	Project name	Objectives of the study	Stakeholders	Findings
[22]	Adolescent health	Adolescent health Study	To provide insights into the policy environment for addressing adolescent mental health in India	Technical Advisory Group (TAG) members, senior researchers, United Nations Fund for Population Activities (UNFPA) representatives	Stakeholder engagement revealed policy gaps in implementing adolescent mental health in India
[23]	Palliative or oncology	Palliative Care Project	To enhance palliative care engagement in intensive care units (ICUs)	Intensive care and palliative care physicians	Reduced ICU deaths, fewer discharges against medical advice, increased palliative care referrals, and patient and family satisfaction
[24]	Maternal and child health	Collaborative Project in Tripura	The study outlines strategies for implementing the research-based intervention, mPower Heart electronic Clinical Decision Support System (e-CDSS), at state-wide health facilities	Project team, government officials, nurses, medical officers, chief medical officers, district coordinators	Stakeholder engagement and various strategies led to a significant population-level impact, enhancing non-communicable disease care in Tripura’s government health facilities
[25]	Public health	Public health initiative	To develop recommendations on the engagement of different stakeholders and disadvantaged populations in priority-setting	Policymakers, clinicians and members from public health agencies	Stakeholder engagement resulted in the creation of recommendations for transparent priority-setting processes that systematically address conflicting interests
[26]	Nutrition	IYCN Programs Analysis	The aim of this study was to: (i) capture stakeholder networks in relation to funding and technical support for IYCF policy across five countries in South Asia (i.e. Sri Lanka, India, Nepal, Bangladesh and Pakistan) and (ii) understand how stakeholder networks differed between countries and identify common actors and their patterns in network engagement across the region	Government stakeholders from South Asian countries	Stakeholder engagement offered vital insights into funding and technical support networks for IYCF practices, notably benefiting government stakeholders through collaborative efforts
[27]	Oncology	ICANTREAT Initiative	To understand current processes, develop improvement strategies and promote the utilization of breast cancer screening and diagnostic facilities	Physicians, surgeons, therapists, community leaders, program officers, health professionals, survivors	The workshop provided crucial insights for developing practical, achievable and sustainable interventions such as a clinical care pathway, marking the start of the ICANTREAT initiative and a Community of Expertise
[28]	Public health	Avoidable Blindness project	To develop models of care for reducing avoidable blindness involved various stakeholders, including	Professional experts, public health personnel, government staff, nongovernmental organization (NGO) representatives	The project successfully integrated screening and treatment for retinopathy of prematurity and diabetic retinopathy into the public health system through a partnership approach, ensuring sustainability and promoting comprehensive eye care
[29]	Public health	Tuver Demonstration Project	To demonstrate diverse stakeholders’ engagement with expertise in health, business, public health, engineering and medicine	Stakeholders from the health, business, public health, engineering and medicine sectors	Despite challenges, involving local staff and intermediaries effectively bridged gaps and enhanced the project’s long-term sustainability, leading to improved health and wellbeing within the marginalized community
[30]	Communicable diseases	Pediatric TB Testing Initiative	To catalyse the adoption of upfront Xpert testing in paediatric presumptive tuberculosis (TB)cases in major Indian cities	Healthcare providers, facilities, organizations	Stakeholder engagement showed notable increases in provider participation and diagnostic uptake, underscoring the vital role of proactive engagement in scaling up rapid diagnostics and enhancing healthcare delivery outcomes
[3]	Public health	Roundtable Discussion on Implementation Research	To translate research findings into actionable policy recommendations and foster frequent partnerships to facilitate capacity-building for evidence-based decision-making	Rural district medical officers, Ministry of Health experts, policymakers	Stakeholders highlighted the benefits of engagement in research and called for continued dialogue and capacity-building initiatives to strengthen collaboration in the future
[31]	Health equity	Health Equity Research Agenda	To identify research gaps, develop a comprehensive agenda and prioritize immediate research needs, aligning with key stakeholders’ priorities and capturing diverse perspectives	Researchers, policymakers, activists	The research agenda’s strength lies in its inclusive development process, gathering inputs from diverse stakeholders to understand health inequities in India comprehensively and identify actionable research priorities
[32]	Maternal and Child Health	Sundarbans study	To understand the linkages between these stakeholders and enhance their capacity to utilize evidence for actionable steps	Mothers, informal healthcare providers, stakeholders from the region	Stakeholder engagement had a positive impact on deepening understanding and fostering collaboration
[33]	Mental health	Mental Health Services Scaling study	To evaluate the stakeholders’ interests, influence, positions, and potential impact on mental health service scaling-up across Ethiopia, India, Nepal, South Africa and Uganda	Policymakers, donors, specialists, media, universities, others	Qualitative stakeholder analysis proved crucial in engaging stakeholders and identifying strategies to stimulate research demand amongst policymakers and practitioners
[34]	Rural healthcare	Rural Healthcare Campaign study	To train the accredited social health activists (ASHAs), providing technical support to state governments and implementing a mass media campaign to increase oral rehydration solution (ORS) and combined ORS and zinc use amongst children aged 2–59 months with diarrhoea	Private rural healthcare providers, ASHAs, state governments, caregivers	Stakeholder engagement had a positive impact on involving public and private providers alongside mass media campaigns in enhancing ORS and zinc utilization for managing diarrhoea
[35]	Public health	City Governance study	To assess cause-and-effect analysis and workshop synthesis to develop systemic change hypotheses and leverage opportunities	City officials, workers from Health and Family Welfare, Women and Child Development, Education, Indore Municipal Corporation, ISCDL, Madhya Pradesh Pollution Control Board	Stakeholder engagement identified three leverage opportunities and co-created seven coherent actions, aiding Indore's transition to a healthier, more equitable state through effective collaboration and active participation in systems thinking workshops
[36]	Maternal and child health	IYCF Policy Development study	To analyse stakeholders’ linkages, influences and goals related to Infant and Young Child Feeding (IYCF) policy, thereby identifying influential actors and their impact on policy and program decisions	Policymakers, program managers at national and state levels	The stakeholder engagement process uncovered strengths in India’s IYCF policy environment, such as integrating IYCF policies into health and child development agendas and guidelines at national and state levels
[37]	Public health	Multidisciplinary Health study	To comprehensively assess existing pregnancy care networks, challenges and opportunities by discussing infrastructural issues, mapping stakeholder interactions and detailing daily activities in the field related to pregnancy care	Academics, researchers, community stakeholders	Stakeholder engagement offered valuable insights for future research and designing digital technologies to improve pregnancy care in low and middle-income countries
[38]	Mental health	Mental Health study	To explore the impact and facilitators of the green skills program (GSP), face-to-face interviews were conducted for on-campus participants, and telephone interviews were conducted for others due to coronavirus disease 2019 (COVID-19) restrictions	Patients, caregivers, staff, professionals, NGO personnel, supervisors	Stakeholders noted various patient improvements, attributing them to the program’s peer learning, teamwork, motivating teaching style and incentives, which boosted engagement, social skills and overall wellbeing
[39]	Adolescent health	RKSK Health Workers study	The study investigates the impact of COVID-19 on the implementation of the National Adolescent Health Programme’s peer education program, the repurposing of health workers and its impact on adolescents’ health and development	Health workers, peer educators	Stakeholder engagement stressed the crucial role of peer educators (PEs), supported by community health workers, in meeting community needs during a pandemic
[40]	Public health	Tobacco Cessation Integration study	To assess the feasibility of integrating a tobacco cessation package with data analysis facilitated by the framework method	Medical officers, counsellors, nurses, program officers	Healthcare providers significantly impacted tobacco cessation support, advocating for tailored counselling methods and suggesting adaptations to maintain provider motivation, alongside identifying facilitators such as inter-programmatic referral systems and politico-administrative commitment
[41]	Public health	Polio Eradication Efforts study	The study uses a conceptual partnership framework, specifically focussing on the core group polio project (CGPP) partnership in India and stakeholder collaboration for polio eradication	United Nations International Children's Emergency Fund (UNICEF), WHO, Rotary International, Indian government	Stakeholder partnerships, facilitated by mechanisms such as the social mobilization working group and coordination meetings, are pivotal for the success of polio eradication efforts in India
[42]	Community health	Community Health study	To ensure sustained involvement and comprehensive analysis of stakeholders’ perspectives in adopted strategies during the COVID-19 pandemic	Patients, caregivers, community leaders, health workers, providers, managers, policymakers, leaders	Partners addressed stakeholders’ needs through diverse communication channels, effectively acknowledging and responding to COVID-19 risks, increased workloads and resource allocation
[43]	Community support	Community Support study	The review focusses on the identification, accountability mapping, support system and engagement process of urban poor populations in LMICs during the COVID-19 pandemic	Volunteers, community organizations, civil society, support entities	The interest–influence matrix revealed that specific stakeholders, despite having high interest, had less influence, emphasizing the need for their recognition and engagement
[44]	Healthcare development	Healthcare Development study	To ensure compatibility with the local context and optimize resource utilization to eradicate communicable and non-communicable diseases	Healthcare providers such as general physicians, nurses, technicians, physiotherapists, counsellors, data entry operators	Training objectives were tailored to stakeholder feedback, guiding the training of state and district program managers to improve care services for diverse communities
[45]	Universal health coverage	Universal Health Coverage Commission study	To develop theory of change (ToC) frameworks and future considerations, including citizens’ consultations and additional stakeholder engagement	Commission members, external experts, fellows, and additional participants representing UHC sectors	Emphasizing ongoing citizen consultations and engagement with administrative leaders is vital to refining the ToC and aligning the strategy with diverse stakeholder needs, promoting a more equitable and effective healthcare delivery system in India

### Quality appraisal of included studies

In total, nine of the included studies were descriptive and could not be included in the quality appraisal process [3, 22, 25, 29, 31, 32, 41–43]. Of the six qualitative studies, two were rated moderate quality [35, 36] and four were rated high [33, 38–40] as per the CASP checklist (Table 3). The remaining 10 studies were rated using the MMAT checklist; 6 were rated moderate [24, 27, 28, 30, 34, 44] and 4 were rated high quality [23, 26, 37, 45] (Table 4). Thus, 25 studies were included in the final synthesis.

**Table 3:** Quality appraisal of included studies using the CASP checklist

Author and year	Was there a clear statement of the aims of the research?	Is a qualitative methodology appropriate?	Was the research design appropriate to address the aims of the research?	Was the recruitment strategy appropriate to the aims of the research?	Was the data collected in a way that addressed the research issue?	Has the relationship between the researcher and participants been adequately considered?	Have ethical issues been taken into consideration?	Was the data analysis sufficiently rigorous?	Is there a clear statement of findings?	How valuable is the research?
Makan et al., [33]	Yes	Yes	Yes	Yes	Yes	Yes	Yes	Yes	Yes	Yes
Bakhtawar et al., [35]	Yes	Yes	Yes	Yes	Yes	No	No	Yes	Yes	Yes
Puri et al., [36]	Yes	Yes	Yes	Yes	Yes	No	No	Yes	Yes	Yes
Roy et al., [38]	Yes	Yes	Yes	Yes	Yes	Yes	Yes	Yes	Yes	Yes
Arora et al., [39]	Yes	Yes	Yes	Yes	Yes	Yes	Yes	Yes	Yes	Yes
Bhatt et al., [40]	Yes	Yes	Yes	Yes	Yes	Yes	Yes	Yes	Yes	Yes

**Table 4:** Quality appraisal of included studies using the MMAT checklist

Author and year	Clear research question	Appropriate qualitative approach	Adequate data collection	Substantiated findings	Well-supported interpretations	Overall quality
Theory of change studies (*n* = 2) and mapping (*n* = 4)						
Chaudhuri et al., [45]	Yes	Yes	Yes	Yes	Yes	High
Rao et al., [23]	Yes	Yes	Yes	Yes	Yes	High
Bagalkot et al., [37]	Yes	Yes	Yes	Yes	Yes	High
Bhatt et al., [44]	Yes	Yes	Yes	Yes	No	Moderate
Uddin et al., [26]	Yes	Yes	Yes	Yes	Yes	High
Kathrikolly et al., [27]	Yes	Yes	Yes	Yes	No	Moderate

Author and year	Clear research question	Appropriate sampling	Validated measurements	Suitable statistical analysis	Discussion of limitations	Overall quality
Quantitative studies (*n* = 1)						
Lam et al., [34]	Yes	Yes	Yes	Yes	No	Moderate

Author and year	Clear research question	Appropriate mixed-methods design	Adequate data collection	Effective integration of findings	Discussion of limitations	Overall quality
Mixed method studies (*n* = 3)						
Jindal et al., [24]	Yes	Yes	Yes	Yes	No	Moderate
Gudlavalleti et al., [28]	Yes	Yes	Yes	Yes	No	Moderate
Raizada et al., [30]	Yes	Yes	Yes	Yes	No	Moderate

### Type of stakeholders

**Fig. 2:**
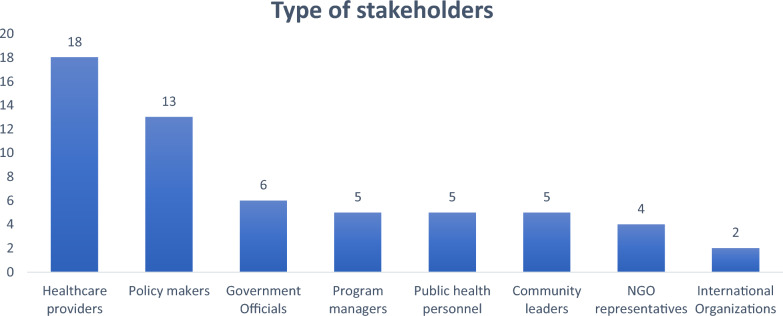
Distribution of stakeholder types in selected studies; *N* = 25

The 25 studies reviewed have diverse stakeholders in the healthcare field. This included healthcare providers such as nurses and doctors, policymakers, government officials, program managers, community leaders, NGOs and international organizations such as UNFPA, UNICEF, and WHO (Fig. 2). These stakeholders played various roles, from decision-making and coordination of healthcare programs to local engagement and providing global health expertise.

Amongst the 25 studies, the most commonly involved type of stakeholders were healthcare providers, including nurses, medical officers, physicians and health workers, who appeared across multiple studies (*n* = 18). Other frequent stakeholders included policymakers (*n* = 13), government officials (*n* = 6) and program managers (*n* = 5), reflecting significant involvement from both healthcare professionals and decision-makers. Additionally, public health personnel (*n* = 5), NGO representatives (*n* = 4) and community leaders (*n* = 5) were commonly engaged, often collaborating with international organizations (*n* = 2) such as UNFPA, UNICEF and WHO. The studies highlighted various stakeholders from sectors such as health, public health and education, emphasizing cross-sectoral engagement in public health initiatives.

### Diverse approaches to stakeholder engagement

Two studies integrating policy reviews with semi-structured interviews highlight the role of technical coordination units and structured change management in securing stakeholder buy-in and large-scale implementation [23, 25]. However, administrative structures alone may not ensure sustainable, community-driven change. Three studies emphasize focus group discussions and workshops as effective platforms for collaborative decision-making in priority-settings [24, 26, 28]. Whilst these participatory approaches enhance inclusivity, they risk dominant voices overshadowing marginalized perspectives, underscoring the need for structured facilitation to balance power dynamics.

Three studies using the Net-Map method effectively aligned stakeholder efforts for policy advocacy in infant and young child feeding (IYCF) [27, 37, 38], though its impact may be limited without policy interventions. Another study employed participatory decision-making for advocacy and policy development, enhancing engagement and relevance [29]. Whilst this approach ensures policies reflect local needs, sustained stakeholder commitment is crucial to prevent fragmentation and loss of momentum.

The business of humanity (BoH) approach, integrating stakeholder engagement with community power-sharing, offers strategic benefits [30], though its scalability in resource-limited settings remains uncertain. Systematic outreach and education foster participation and sustainability [31] but require accountability mechanisms and long-term knowledge transfer for lasting impact. Of the four studies, Facilitating direct communication between researchers and decision-makers has been instrumental in translating research findings into actionable policy recommendations [5, 32, 34, 43] but needs sustained collaboration and institutional backing. The theory of change (ToC) framework fosters stakeholder-aligned interventions[45], though its success depends on adaptive learning to meet evolving needs (Table 5).

**Table 5:** Comparative analysis of stakeholder engagement approaches

Author and year	Location	Approach	Key stakeholder role	Effectiveness and challenges
[23]	India	Policy review and semi-structured interviews	Contributed expertise and identified field challenges	Ensured policy relevance but required broader stakeholder inclusion
[24]	Karnataka	Virtual workshops	Developed research frameworks collaboratively	Risk of dominant voices limiting inclusivity
[25]	Tripura	Change management, training programs	Implemented interventions at health facilities	High buy-in but dependent on administrative support
[26]	Hyderabad	Focus groups, priority-setting	Ensured ethical and equity considerations	Effective but required structured facilitation
[27]	Bangladesh	Net-Map method	Policy funding and technical support	Mapped networks effectively but lacked policy leverage
[28]	Karnataka	Co-design multistakeholder workshops	Informed discussions on breast cancer care	Facilitated engagement but required long-term commitment
[29]	Gujarat, Karnataka, Kerala, Telangana	Participatory approach	Integrated screening and treatment	Enhanced engagement but faced sustainability challenges
[30]	Gujarat	BoH approach	Community leadership and power-sharing	Strategic but needs scalability validation
[31]	India	Systematic outreach, education	Engaged healthcare facilities	Effective but required sustained follow-up
[32]	Kerala	Evidence synthesis, consultations	Addressed health inequities	Holistic but needed continued advocacy
[33]	India, Uganda	Network mapping, document reviews	Applied participatory impact analysis	Useful for systems analysis but complex to implement
[34]	Multi-country	Strategy formulation	Scaled-up mental health interventions	Broad impact but resource-intensive
[45]	India	Theory of change (ToC)	Developed stakeholder-aligned frameworks	Iterative but required adaptive learning

### Stakeholder engagement methods in shaping healthcare policies and practices

Of the four studies, participatory approaches, such as facilitated workshops and multisectoral discussions, have fostered collaboration and inclusive healthcare initiatives [24, 28, 29, 38]. However, despite their strengths, these methods are susceptible to dominant voices overshadowing marginalized perspectives. The other four studies used the Net-Map method, which visualizes stakeholder relationships and has clarified roles and improved coordination, leading to more accountable decision-making [27, 31, 33, 37], and whose impact depends on sustained policy interventions and continuous engagement. 

### Impact on policy implementation and crisis response

Of the two studies, evidence generated from stakeholder engagements has provided policymakers with a clearer understanding of stakeholder dynamics, enabling more informed and adaptable decision-making, particularly during crises such as the COVID-19 pandemic [28, 37]. Whilst virtual platforms improved accessibility, digital literacy and technological disparities remain barriers, necessitating targeted capacity-building for effective participation.

**Fig. 3:**
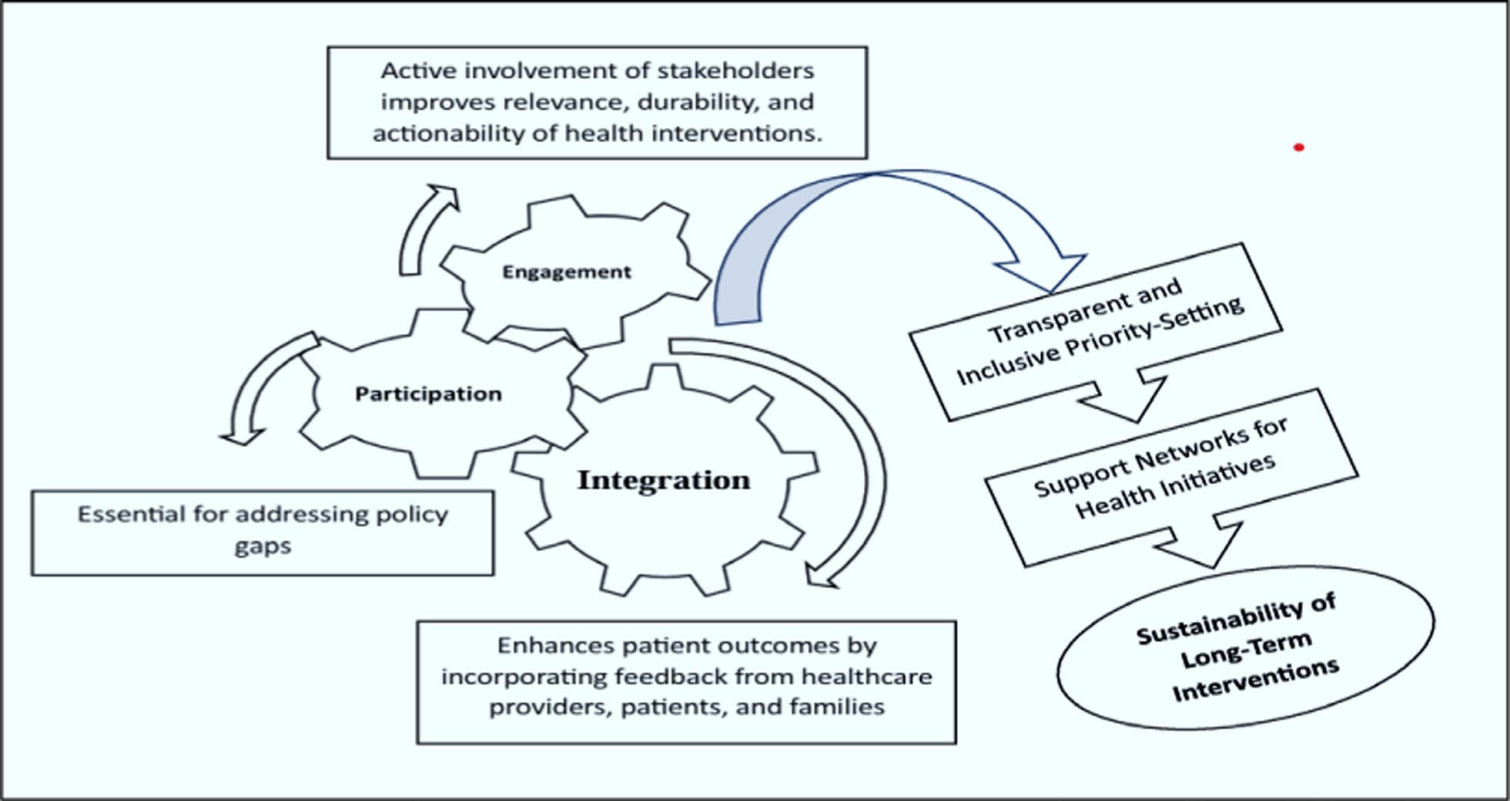
Framework for stakeholder involvement in enhancing healthcare policy and practices

The diagram illustrates a cyclical process for stakeholder engagement in health interventions, emphasizing the integration of engagement and participation to address policy gaps and improve patient outcomes (Fig. 3).

### Expanded applicability in “hard-to-reach” populations

Two studies identified gaps in adolescent mental health policy and non-communicable disease care, highlighting stakeholder engagement’s role in policy development and intervention integration for improved state-level healthcare strategies [22, 24]. Another two studies demonstrated how engaging local actors, including community members and informal providers, strengthens healthcare systems, enhances service delivery and ensures long-term sustainability in marginalized communities [29, 32]. Two studies emphasized targeted interventions’ impact on diagnostic uptake and treatment adherence, with one improving early TB detection and the other increasing ORS and zinc utilization through structured engagement [30, 34]. Another set of studies showcased how peer learning, teamwork and incentives enhance patient engagement and social wellbeing, whilst the Community Health study leveraged diverse communication channels to maintain healthcare responsiveness during COVID-19 [38, 42]. Lastly, one study highlighted the need to recognize overlooked stakeholders in urban poor communities, using an interest–influence matrix to reveal engagement gaps and the necessity of structured involvement for sustained impact [43].

The transparent and inclusive involvement of diverse stakeholders in priority-setting procedures highlights the need to incorporate different socioeconomic groups to create comprehensive healthcare policies [3, 23, 25, 27]. The research findings also emphasized the vital function of national and international institutions in furnishing financial and technical assistance, both of which are important for expanding health-related endeavours [33, 36, 39]. Stakeholder engagement, capacity building and strategic partnerships are critical in extending the reach and impact of health interventions, ensuring that even the most disadvantaged populations receive adequate care and support.

### A strategy for collaborative success and impactful decision-making

**Fig. 4:**
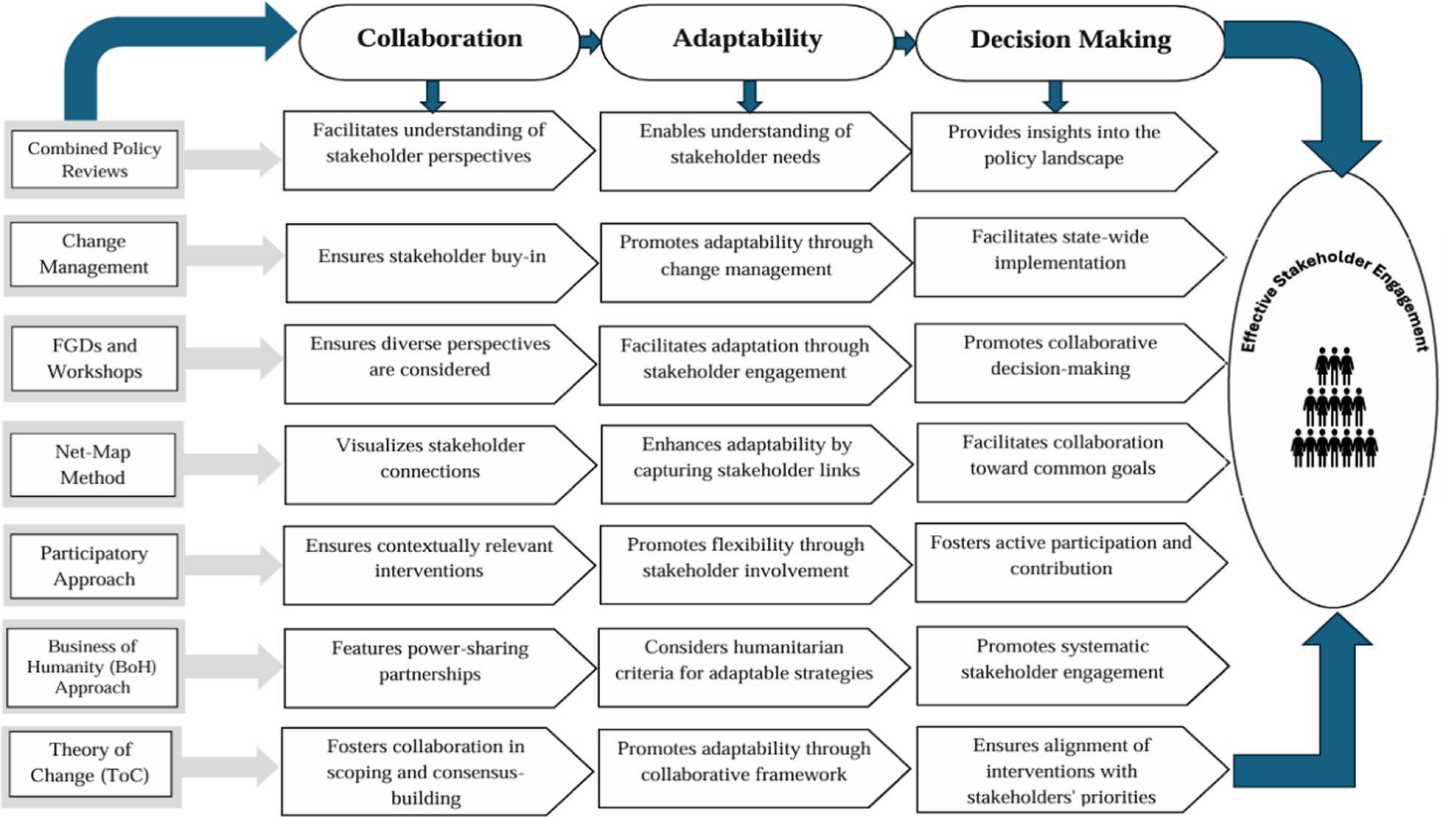
Context and process of stakeholder engagement

The stakeholder engagement process is a dynamic blend of collaboration, adaptability and decisive action to foster comprehensive participation and deliver impactful outcomes. Leveraging semi-structured interviews, policy reviews, focus group discussions and the Net-Map technique, this approach captures diverse perspectives, visualizes stakeholder connections and facilitates informed decision-making [22, 24, 26, 30, 33, 34, 36, 42, 43]. Embracing participatory methodologies and change management strategies ensures the implementation of adaptable interventions with genuine stakeholder support [3, 25, 29, 32, 35, 37, 44, 45]. Emphasizing power-sharing and alignment with stakeholders’ needs, frameworks such as the business of humanity and theory of change and systematic outreach and direct communication pave the way for sustainable, evidence-based decisions [31, 33, 35, 40–43, 45] (Fig. 4).

### Barriers and challenges

The two studies reported that governance fragmentation hindered stakeholder engagement, making priority-setting difficult and leading to inconsistent health policy implementation [25, 31]. Coordination challenges amongst multiple stakeholders affected funding allocation and program effectiveness across different regions [26, 36]. The other two studies highlighted the need for improved coordination to optimize healthcare resources and ensure sustainable health coverage [44, 45]. Youth engagement remains a significant gap, where young people’s perspectives were overlooked in adolescent health programs [22, 39]. Similarly, another study found that youth voices were largely absent from policy discussions, affecting service accessibility and effectiveness.

Cultural differences, varying awareness levels and inconsistent healthcare infrastructure posed significant barriers to integrating palliative care into ICUs [23]. Another study reported systemic barriers, including resistance from healthcare providers and logistical constraints, affected early cancer screening and intervention efforts [27]. Another one reported task shifting to nurses and non-specialist providers in managing non-communicable diseases faced resistance due to insufficient training, lack of support and administrative hesitations [24]. The other two studies found ensuring inclusivity in priority-setting required balancing diverse stakeholder expectations whilst addressing power dynamics [35, 37]. It is reported that managing conflicting interests amongst policymakers, public health agencies and clinicians was challenging when developing transparent priority-setting recommendations [25].

Socioeconomic disparities impacted stakeholders’ ability to participate in decision-making, with less influential groups struggling to assert their needs [43]. Differences in stakeholder resources affected engagement, particularly in ensuring provider motivation and sustainable intervention delivery [40]. Reliance on external funding made implementation vulnerable to policy and funding shifts, necessitating stronger local capacity-building efforts [26]. Ensuring long-term sustainability of health interventions depended on effective integration within existing public health systems [28].

## Discussion

The reviewed studies provide valuable insights into stakeholder engagement, strategies for stakeholder engagement, highlighting its potential benefits and inherent challenges. Stakeholder engagement in healthcare research is crucial for the successful implementation and sustainability of health interventions [24, 28–30, 41]. Effective stakeholder engagement ensures that health interventions align with the needs and expectations of diverse populations, a principle widely recognized in global healthcare systems. For instance, integrating palliative care into intensive care unit (ICU) settings demonstrated long-term benefits, such as reduced ICU deaths and improved patient satisfaction [23]. Similar international initiatives have shown that stakeholder-driven palliative care models improve patient-centred outcomes and resource allocation, reinforcing the need for inclusive engagement [46, 47]. Engaging stakeholders such as healthcare providers, patients and families in designing and implementing these interventions ensures they are contextually relevant and more likely to be effective.

Active stakeholder engagement with diverse stakeholders enhances healthcare research quality and relevance by developing standardized outcome measures and ensuring comprehensive, inclusive and effective healthcare strategies [48, 49]. International models, such as the WHO’s participatory governance frameworks, emphasize multisectoral collaboration to co-design health interventions. The reviewed studies also highlight the importance of engaging diverse socioeconomic groups to capture various perspectives. For example, the mPower Heart e-CDSS program leveraged task shifting to involve nurses in non-communicable disease (NCD) management, demonstrating the value of inclusive capacity-building initiatives [24]. Similarly, workshops and priority-setting exercises that included policymakers, clinicians and the public were crucial for co-designing sustainable health interventions [25, 31]. These findings align with international best practices, such as the UK’s INVOLVE framework and the U.S. Patient-Centered Outcomes Research Institute (PCORI), emphasizing patient and community involvement in research design and decision-making [50, 51].

Many studies highlight key barriers to stakeholder engagement in healthcare research [22–28, 31, 35–37, 39, 40, 43–45]. Globally, stakeholder engagement in healthcare research is hindered by fragmented intersectoral collaboration, stigma in mental health settings and misaligned regulatory policies that hamper innovation and adoption [52–54]. Similar to that in the current review, Governance fragmentation and weak intersectoral coordination hinder effective healthcare policy implementation in India. In contrast, limited youth engagement in policy discussions reduces the relevance of health programs. Additionally, reliance on external funding threatens sustainability, highlighting the need for stronger local engagement strategies.

Addressing these barriers requires strategic planning and resource allocation. The studies suggest several approaches to enhance stakeholder engagement [23–34, 45]. Transparent and inclusive priority-setting ensures diverse voices are considered, leading to equitable healthcare strategies. Innovative methods such as Net-Map enhance stakeholder collaboration, whilst local intermediaries improve accessibility in rural areas. The I-STEM framework, successfully implemented in nine foreign countries, provides a structured approach to overcoming stakeholder engagement barriers through strategic planning and resource allocation [55]. Moreover, the involvement of governmental and international organizations, such as UNICEF and WHO, plays a crucial role in providing financial and technical support, ensuring the sustainability and scalability of health intervention.

## Limitations of the review

Despite its contributions, this review has limitations. First, the studies included exhibit methodological heterogeneity, making direct comparisons challenging. Second, reliance on published literature may introduce publication bias, as unpublished but relevant stakeholder engagement initiatives remain unexamined. Third, whilst many studies highlight engagement successes, fewer focus on failed interventions, limiting a balanced understanding of challenges. Future research should adopt mixed-method approaches to provide a more nuanced analysis of stakeholder engagement effectiveness.

## Conclusions

Effective stakeholder engagement in healthcare research is essential for developing and implementing contextually relevant, sustainable and equitable interventions. The insights from the reviewed studies highlight the importance of strategic and inclusive approaches to stakeholder engagement, ultimately fostering improved health outcomes and system resilience. The reviewed studies emphasize strategic and inclusive engagement approaches, ultimately fostering improved health outcomes and system resilience. In the Indian context, enhancing stakeholder engagement requires integrating digital tools for consultations and training, institutionalizing standardized engagement frameworks, strengthening capacity-building efforts – particularly in rural and underserved areas – and promoting cross-sector collaborations between government agencies, private entities and community organizations.

## Future research directions

Future studies should focus on:

1. *Exploring Digital Engagement Methods* Examining the role of mHealth apps and virtual platforms in stakeholder engagement.
2. *Youth Involvement in Healthcare Research* Investigating strategies to increase youth participation in policy development and health initiatives.
3. *Evaluating Longitudinal Impact* Conducting longitudinal studies to assess the sustained impact of stakeholder engagement on policy and health outcomes.

By implementing these strategies, Indian healthcare research can foster more effective and sustainable stakeholder engagement, ultimately improving health outcomes nationwide.

## Data Availability

No datasets were generated or analysed during the current study.

## Abbreviations

CASP: Critical Appraisal Skills Programme
MMAT: Mixed methods appraisal tool
